# Stereotactic radiation therapy for oligometastatic esophagogastric adenocarcinoma: outcome and prognostic factors

**DOI:** 10.1259/bjr.20220771

**Published:** 2023-03-03

**Authors:** Davide Franceschini, Maria Ausilia Teriaca, Luciana Di Cristina, Veronica Vernier, Lorenzo Lo Faro, Ciro Franzese, Tiziana Comito, Elena Clerici, Luisa Bellu, Luca Dominici, Ruggero Spoto, Maria Massaro, Piera Navarria, Marta Scorsetti

**Affiliations:** 1 Department of Radiotherapy and Radiosurgery, IRCCS Humanitas Research Hospital, Via Manzoni, Rozzano, Milan, Italy; 2 Department of Biomedical Sciences, Humanitas University, Via Rita Levi Montalcini, Pieve Emanuele, Milan, Italy

## Abstract

**Objective:**

The aim of this study was to evaluate clinical results and prognostic factors in a cohort of patient with oligometastatic esophagogastric adenocarcinoma treated with stereotactic radiation therapy (SRT).

**Methods:**

This retrospective study included patients affected by 1–3 metastases treated with SRT from 2013 to 2021. Local control (LC), overall survival (OS), progression-free survival (PFS), time to polymetastatic dissemination (TTPD) and time to systemic therapy change/initiation (TTS) were evaluated.

**Results:**

Between 2013 and 2021, 55 patients were treated with SRT on 80 oligometastatic sites. Median follow-up was 20 months. Nine patients had local progression. 1 and 3 years LC was respectively 92 and 78%. 41 patients experienced further distant disease progression, median PFS was 9.6 months, 1 and 3 years PFS was respectively 40 and 15%. 34 patients died, median OS was 26.6 months, 1 and 3 years OS was respectively 78 and 40%. During follow-up, 24 patients changed or initiated a new systemic therapy; median TTS time was 9 months. 27 patients experienced poliprogression, 44% after 1 year and 52% after 3 years. Median TTPD was 8 months. The best local response (LR), tyming of metastases and PS were related with prolonged PFS on multivariate analysis. LR was correlated with OS at multivariate analysis.

**Conclusion:**

SRT represents a valid treatment for oligometastatic esophagogastric adenocarcinoma. CR correlated with PFS and OS, while metachronous metastasis and a good PS correlated with a better PFS.

**Advances in knowledge:**

In selected gastroesopagheal oligometastatic patients, SRT can prolong OS Local response to SRT, metachronous timing of metastases and better PS improve PFS.

Local response correlates with OS.

## Introduction

Esophageal and gastric cancer represents a major epidemiological issue and a relevant cause of cancer death.^1^ Despite advancements both in local and systemic treatment, approximately half of patients still relapse after primary curative treatment, particularly in node positive disease, and one-third of patients has already metastatic disease at diagnosis.^2^ Prognosis of Stage IV disease is generally bad, ranging from approximately 4 months with best supportive care to 12 months with chemotherapy.^3^

Oligometastatic disease (OMD) is a distinct clinical state between localized disease and widespread metastatic disease, as first theorized in 1995,^4^ by definition histology agnostic. Recent prospective trials validated the use of local ablative treatments (LATs) including stereotactic radiation therapy (SRT) for these patients, with most data focused on lung, prostate and colorectal cancer.^5–9^ For esophagogastric cancer, randomized control trials are ongoing and the available evidences are derived from few non-randomized studies^10,11^ and retrospective series, mainly of surgical metastasectomy. A recent systematic review and meta-analysis, done by the oligometastatic esophagogastric cancer (OMEC) consortium, identified 16 studies reporting survival outcomes in patients receiving also LAT as part of their therapy.^12^ The same group analyzed 97 studies to derive a shared definition of oligometastatic esophagogastric cancer. The authors’consensus defined as OMD disease one organ with ≤3 metastases or one extraregional lymph node station; on the contrary, no consensus was reached about treatment strategies for this disease in a parallel project of the same consortium.^13^

Moving from this definition of OMD esophagogastric cancer, we conducted this analysis on our patients, focusing on adenocarcinoma patients, to evaluate the impact of SRT in this scenario. We evaluated survival outcomes (overall survival [OS], progression-free survival [PFS], local control [LC]) and possible patients and treatment factors related to prognosis. We also estimated time to polymetastatic dissemination (TTPD) in patients experiencing a further disease progression and time to systemic therapy change/initiation (TTS), as novel endpoints of interest in the OMD setting.

## Methods and material

From an institutional database collecting data of oligometastatic patients treated with SRT from 2013, we extrapolated data on esophagogastric adenocarcinoma patients affected by less than three metastases in one distant organ or one organ and one nodal station. For each patient, the following information were collected: date of birth; sex; date of primary cancer diagnosis; primary cancer site (stomach, esophagus or gastroesophageal junction GEJ); adenocarcinoma subtype; treatment of primary cancer; clinical or pathological stage of primary cancer; date of Stage IV diagnosis; imaging performed for OMD diagnosis; comorbidities; performance status (PS) according to Eastern Cooperative Oncology Group (ECOG); smoking habits; timing of metastases occurrence (synchronous metastases were defined as occurring <6 months from primary diagnosis); type of oligometastases according to ESTRO-EORTC classification^14^ ; treatments received for Stage IV disease before SRT; date, dose and number of fractions of SRT; number and site of irradiated metastases; concomitant systemic therapies.

The study was conducted with the approval of institutional review boards, and each patient signed an informed consent at SRT time to use data for future research.

Patients were all treated with SRT with volumetric modulated arc therapy in its rapid arc form (VMAT-RA). Different dose and fractionation schedules were employed according to site and size of the lesion(s) and to regional organs at risk (OARs) dose constraints. A balance to maximize efficacy while minimizing toxicity was pursued in all cases.

Biologically equivalent dose (BED) was calculated assuming a α/β ratio of 10 Gy. In case different metastases were treated with different doses in the same patient, the lowest BED per patient was used for the analysis.

Patients were regularly followed-up, generally with CT scan and or MRI 3 months after SRT and a PET/CT after 6 months. Thereafter, follow-up was scheduled according to response, disease status and international guidelines. Response to radiotherapy was scored according to European Organization for Research and Treatment of Cancer Response Evaluation Criteria In Solid Tumours (EORTC-RECIST) criteria v. 1.1^15^ and according to PET Response Criteria in Solid Tumors (PERCIST).^16^ Acute and late toxicities were recorded and scored according to Common Terminology Criteria for Adverse Events (CTCAE) v. 5.0.

### Statistical analysis

Last day of SRT was utilized for survival analysis. OS was calculated until last follow-up visit or death from any cause, whatever it occurs first. LC was calculated until last follow-up visit or local recurrence. PFS included as events both local and further distant recurrence whatever occurs first. Further distant progression was classified as oligo- or polyprogression according to number of new/progressing metastases and number of involved organs (one–three new/progressing metastases in only one organ or one organ and one nodal station) and date of polyprogression was used to estimate the TTPD. Subsequent systemic therapies received by patients were recorded and the date of change/initiation of the new therapy was used to estimate TTS.

All event occurrences were analyzed with the Kaplan–Meier method and the long-rank test was used for univariate analysis (UVA) to compare subgroups. Further, factors with *p*-value < 0.05 obtained from UVA were included in the multivariate analysis (MVA) to search for independent predictive factors. Statistical analyses were performed using MedCalc for Windows v. 20.110 (MedCalc Software, Ostend, Belgium).

## Results

### Patients and treatment characteristics

Between 2013 and 2021, 55 patients were treated with SRT on 80 oligometastatic sites. Median age was 69 years (range 43–91). Most frequently, patients were treated on nodal metastases (22, 40%), followed by liver (14, 25.45%) and lung lesions (13, 23.64%). Only three patients were treated on one organ and one nodal station, precisely adrenal gland in two cases and liver in the other one. Considering nodal metastases, in all but two cases these were outside the field of a previous RT, if received. In most cases, patients were irradiated for a single metastasis, 2 lesions were simultaneously treated in 13 patients (23.64%) and 3 lesions in 6 cases (10.91%). More than half patients (32, 58.18%) received SRT as upfront therapy, with no other previous systemic treatment for Stage IV disease. Primary tumor, patients and oligometastatic disease characteristics are shown in Tables 1 and 2. Dose and fractionations employed are shown in Table 3. Concomitant drugs were in most cases chemotherapy (5/7) and trastuzumab in the remnant two cases.

**Table 1.** Patient and primary disease characteristics

Characteristics	Individuals	Percentage
***Gender*** Male Female	12 43	21.82% 78.18%
***Presence of comorbidities*** Yes No	41 14	74.55% 25.45%
***PS*** 0 1	31 24	56.36% 43.64%
***Smokers*** Active Ex Never	9 21 25	16.36% 38.18% 45.46%
***Primary tumor site*** Stomach Gastroesophageal junction Esophagus	22 18 15	40% 32.73% 27.27%
***Type of adenocarcinoma*** Tubular Type Others	18 37	32.73% 67.27%
***Neoadjuvant systemic therapy for the primary tumor*** Yes No	25 30	45.45% 54.55%
***Neoadjuvant radiotherapy for the primary tumor*** Yes No	13 42	23.64% 76.36%
***Surgery for the primary tumor*** Yes No	49 6	89.1% 10.9%
***Adjuvant systemic therapy for the primary tumor*** Yes No	15 40	27.27% 72.73%

PS = performance status.

**Table 2.** Oligometastatic disease characteristics

Characteristics	Individuals	Percentage
***Imaging*** CT scan only CT scan+other	13 42	23.64% 76.36%
***Disease-free interval***	493 days (median)	0–2497 (range)
***Oligometastatic type*** *De novo* synchronous *De novo* metachronous oligorecurrence Repeat oligorecurrence Repeat oligoprogression Induced oligorecurrence Induced oligopersistence Induced oligoprogression	4 26 7 1 13 3 1	7.27% 47.27% 12.73% 1.82% 23.64% 5.45% 1.82%
***Timing of metastases*** Synchronous Metachronous	10 45	18.18% 81.82%
***Number of irradiated lesions*** 1 2 3	36 13 6	65.45% 23.64% 10.91%
***Location of irradiated lesions*** Lung Brain Liver Adrenal gland Nodes Lung+Node Adrenal gland+Node	13 2 14 1 22 1 2	23.64% 3.64% 25.45% 1.82% 40% 1.82% 3.63%
***SBRT BED***	78.75 (median)	45–262.5 (range)
***Concomitant systemic therapy*** Yes No	7 48	12.73% 87.27%

BED = biologically effective dose;SBRT = stereotactic body radiotherapy.

**Table 3.** SBRT dose and fractionations

Dose	Number of fractions	Number of patients (%)
20	1	1
30	3	1
30	5	4
30	6	1
35	5	1
36	3	1
36	6	5
40	4	2
40	5	1
45	6	13
48	4	6
48	6	1
50	10	1
50	5	3
54	3	2
54	6	2
60	3	3
60	6	1
63	6	1
67.5	3	2
75	3	3

SBRT = stereotactic body radiotherapy.

**Figure 1.**
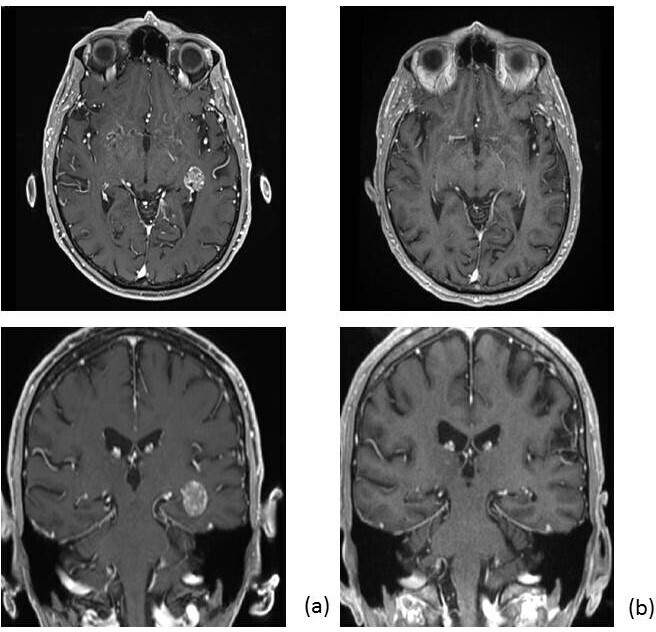
Brain MRI showing a metastases (**a**) from gastric cancer treated with RS, 24 Gy in 1 fraction with complete response at follow-up MRI (**b**). RS, radiosurgery.

During follow-up, we observed as best response 21 complete response (CR); 24 partial response (RP); 8 stable disease (SD) and 2 progressive disease (PD). An example of CR of brain metastases from esophageal cancer is shown in Figure 1.

### Toxicity

Treatment was generally well tolerated. No G3 or higher toxicity was recorded

One patient developed G2 pneumonitis few weeks after lung SRT. As acute side-effects, four other patients reported G1 toxicity (headache G1, gastritis G1, dysphagia G1 and dyspepsia G1). During follow-up, three other patients complained late toxicity (dyspnea G1 in two patients, cough G1 and chest wall pain G1).

Toxicities linked with irradiated site and RT dose and fractionation are shown in Table 4.

**Table 4.** Acute and late toxicity linked with irradiated site(s) and RT dose

Acute toxicity			
**Toxicity**	**Grade**	**Irradiated site**	**RT dose/fractionation**
Headache	G1	Brain	20 Gy/1 fraction
Gastritis	G1	Liver	67.5 Gy/3 fractions
Pneumonitis	G1	Lung	50 Gy/5 fractions
Dysphagia	G1	Thoracic node	30 Gy/5 fractions
Dyspepsia	G1	Liver	54 Gy/6 fractions
Pneumonitis	G2	Lung	48 Gy/4 fractions
**Late toxicity**			
Dyspnea	G1	Lung	50 Gy/10 fractions
Dyspnea	G1	Lung	40 Gy/5 fractions
Cough	G1	Lung	50 Gy/10 fractions
Abdominal pain	G1	Liver	45 Gy/6 fractions

RT = stereotactic body radiotherapy.

### Overall survival

**Figure 2.**
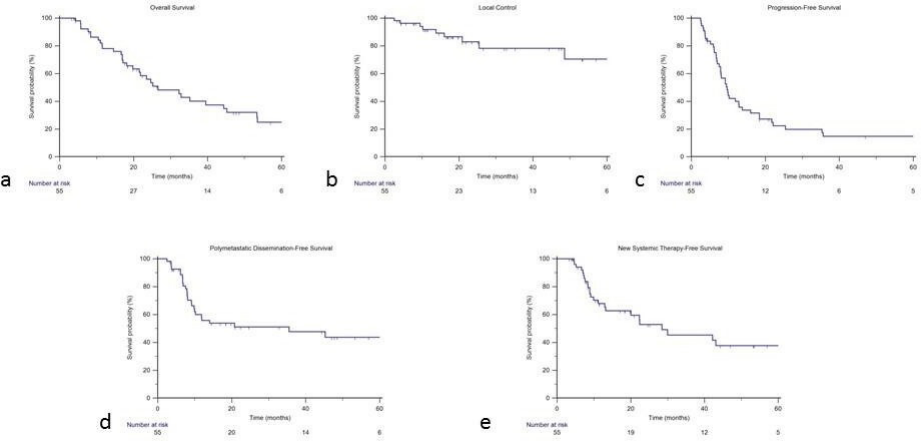
Kaplan–Meier curves for overall survival (**a**), local control (**b**), progression-free survival (**c**), polymetastatic dissemination-free survival (**d**) and new systemic therapy-free survival (**e**).

With a median follow-up of 20 months (range 3–96), 34 patients died (all but three due to neoplastic disease progression). At last follow-up, seven patients had no evidence of disease, five patients were alive with persisting disease in the irradiated lesion(s) and nine patients were alive with further distant metastases. Median OS was 26.6 months, 1, 2 and 3 years OS was respectively 78%, 56% and 40% (Figure 2a).

**Figure 3.**
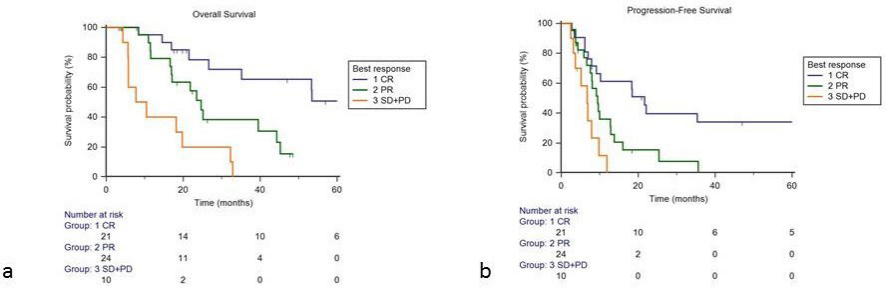
Overall survival (**a**) and progression-free survival (**b**) according to best local response of irradiated lesion(s). CR, complete response; PD, progressive disease; PR, partial response; SD. stable disease.

At univariate analysis, best local response (CR *vs* PR *vs* SD + PD [*p* < 0.0001]) and BED >75 Gy (p 0.0353) correlated with OS. At multivariate analysis, best local response maintained a predictive value, both considering CR compared with PR+SD+ PD (HR 0.28 95% CI 0.11 to 0.75; p 0.01) and comparing SD + PD vs CR+PR (HR 2.99 95% CI 1.29–6.93; p 0.01) (Table 5) (Figure 3a).

**Table 5.** Multivariate analysis of prognostic factors for overall survival and progression-free survival

End point	Variable	HR	95% CI for HR	*p-*value
PFS	Timing of metastases (synchronous *vs* metachronous)	3.07	1.50–6.26	0.002
	ECOG PS (1 *vs* 0)	2.10	1.10–3.97	0.020
	SD + PD *vs* CR + PR	2.36	1.02–5.59	0.040
	CR *vs* RP + SD+PD	0.36	0.17–0.76	0.008
OS	SD + PD *vs* CR + PR	2.99	1.29–6.93	0.010
	CR *vs* RP + SD+PD	0.29	0.11–0.75	0.011

CI = confidence interval; CR = complete response; HR = hazard ratio;OS = overall survival; PD = progressive disease; PFS = progression-free survival; PR = partial response; ECOG PS = Eastern Cooperative Oncology Group performance status; SD = stable disease.

### Local control

During follow-up, nine patients experienced a local progression, after a median time of 13.9 months (range 2.6–36.1). Median LC was not reached. 1 and 3 years LC was respectively 92 and 78% (Figure 2b). At univariate analysis, none of the analyzed parameters correlated with LC. Only one patient was retreated with a further course of SRT.

### Progression-free survival

Further distant progression was the main pattern of failure. Indeed, 41 patients had a further disease diffusion (one only locally, in all other cases distant or distant+local). Median time to distant progression was 8 months (range 2.6–36.1). More than half patients (22,55%) progressed initially again in an “oligo” way. Thirteen patients received a second course of local therapy (SRT or surgery), while 15 of them were treated with systemic therapy. The remnant patients were candidate to best supportive care.

Considering both local and distant progression, whatever occurs first, median PFS was 9.6 months (range 7.9–12.9), 1 and 3-years PFS was respectively 40 and 15% (Figure 2c).

At univariate performance status (*p* 0.02), timing of metastases (*p* 0.0092), DFI > 493 days (*p* 0.0260) and best local response (*p* 0.0003) (LR) correlated with PFS. At multivariate analysis, best LR, PS and timing of metastases maintained statistical significance with a *p*-value < 0.001 (Table 3) (Figure 3b).

### Time to polymetastatic dissemination

As said above, 18 patients experience a polymetastatic dissemination as first event after SRT. During follow up, other 9 patients initially progressing as oligometastatic again developed ultimately a widespread dissemination. Considering these 27 patients, median TTPD was 8 months (range 3–82). Polymetastatic progression free survival was 56% after 1 year and 48% after 3 years (Figure 2d).

### Time to change/initiate new systemic therapy

24 patients change or initiate a new systemic therapy after SRT due to disease progression. Median TTS time was 9 months (range 5–43). New systemic therapy free survival was 68% at 1 year and 45% at 3 years (Figure 2e).

## Discussion

We report a single institution experience on the use of SRT for OMD from esophagogastric cancer, as defined by the OMEC consortium,^12^ with the only exception of three patients that were affected by metastases in a visceral organ and in a single nodal station. Our data confirmed the excellent toxicity profile of SRT, high LC rates (78% at 3 years) and a promising OS (median 26.6 months), longer than the historical prognosis of patients treated with systemic therapy only.^3^ As commonly described in oligometastatic series, the major pattern of failure was further distant dissemination, with a median PFS of 9.6 months, similar to those reported with other histology in prospective Phase II trials.^6,17^

Few data are available regarding esophagogastric cancer, compared to other primary tumors. Most of them come from surgical series or miscellaneous LAT.

It is quite difficult to estimate the proportion of OM patients in esophagogastric carcinoma compared with all Stage IV patients. In the Agamenon surgical registry, the proportion of patients who underwent surgical metastasectomy compared to all patients in the registry with advanced adenocarcinoma of the stomach, distal esophagus, or gastroesophageal junction was only 5%.^2^ A pattern of failure analysis presented in 2020^18^ analyzed 1438 patients, finding that 197 (13.7%) of them were oligometastatic (with a broader definition of the one used in our study). Interestingly, they also found that, of 152 pts who progressed, 91 (60%) had isolated initial site failure, 17% had isolated new site failure, and 23% had both initial and new site failure. Only 11% of patients were treated with RT to metastatic site, *i.e*. this high percentage of original site failure (83%) represents a good rationale to support LAT in this selected population. Indeed, original site failure in our experience occurred only in 16% of cases (experiencing a local relapse), suggesting that the use of SRT at least changed the trajectory of the disease, apart from prolonging survival.

In the Phase II trial by Liu et al,^10^ 34 patients were treated on 40 OMD, defined as 3 or fewer metastases amenable to SRT with primary tumor controlled. All patients were affected by squamous cell carcinoma of the esophagus. Authors reported OS at 1 and 2 years comparable to our (76.2 and 58 *vs*  78.1% and 56%), with a similar absence of relevant toxicity. The median PFS was 13.3 months; the 1- and 2-year PFS rates were 55.9 and 33.8%, respectively. These results are slightly better than ours and probably could reflect the prospective selection of patients, the different histology and disease, and also the fact that half of patients received “adjuvant” chemotherapy in their experience (compared with 13% in our series). Moreover, half of our patients were not pre-treated with systemic therapy before SRT, generally due to comorbidities or advanced age. Anyway, in our opinion, a better integration of local and systemic approaches should be pursued and could be the key to reduce distant failure. Ongoing trials, like the Renaissance trial (NCT02578368) and the ECOG-ACRIN trial (NCT04248452), could better clarify the benefit of this combination, since both consider an “induction” chemotherapy followed by local approach (surgery or radiotherapy) in the experimental arm.

A median OS of 25 months and a median 1-year and 5-year OS rates of 75 and 44% were found by the systematic review of 16 retrospective studies for a total of 740 analyzed patients, including patients treated with all LAT.^12^ The similar OS in our series deserves to be highlighted, considering the unfavorable selection of our patients when compared to surgical candidates. General condition of patients is a well-known prognostic factor in oncology, indeed also in our study a better PS correlated with longer PFS. In any case, these survival data should be taken very carefully due to the significant risk of biases due to the retrospective nature of the studies.

In our analysis, we also tried to identify prognostic factors predictive for outcome, since a key in the oligometastatic world is the selection of patients and the identification of predictive biomarkers. This predictive ability could be even more relevant in an aggressive tumor like esophagogastric cancer with a limited proportion of “true” oligo patients. In surgical patients, Carmona-Bayonas et al^2^ identified the duration of chemotherapy before surgery as correlated to mortality, suggesting that any delay before LAT should be kept at minimum. They also found a survival benefit in HER2+ patients, although this could simply reflect the availability of more effective systemic therapies in this category of patients. In our experience, systemic therapies before or during SRT were not related with survival outcomes.

Still remaining in the surgical scenario, another study^19^ identified as predictive factor for OS at univariate analysis the clinical stage of the primary tumor, type of recurrence (*i.e.* oligometastasis or not), and DFI. However, in the multivariate analysis, only oligometastasis was an independent predictor of OS. Also Ghaly et al^20^ found that time to recurrence was a predictive factor for survival of recurrent esophageal cancer patients after curative esophagectomy. In our experience, a longer DFI was correlated to a longer PFS after SRT, but only at UVA. At the same time, we found a worse PFS for patients affected by synchronous metastases when compared to patients with metachronous occurrence. The same authors also found a better prognosis for patients who underwent surgical resection for nodal metastases compared with visceral. In our study, location of the irradiated site did not influence any outcome.

Li et al^21^ found on multivariate analysis a better PFS correlated with no smoking history and no fistula in a series of metastatic esophageal squamous cell cancer patients. We also analyzed smoking habits of our patients but did not found a statistical correlation, possibly due to the partially different carcinogenesis of esophagogastric adenocarcinoma which was the focus of our work.

We found that BED higher than 75 Gy correlated with OS at univariate analysis. A similar result was reported in the esophagogastric cancer scenario by Li et al^22^ with a dose cut-off of 60 Gy. Also in the general analysis by Hong et al on OMD by various histologies, a BED higher than 75 Gy was included in the model as predictor for survival.^23^ Whenever feasible, an ablative dose is necessary to have a real impact on these patients prognosis. Indeed, we found that the LR of the irradiated lesion(s) strongly correlated with PFS and OS, with the best outcome in patients experiencing a CR. This could be regarded as an indirect proof that an effective local treatment can really have an impact on patients‘ outcome.

Comparing our results with those reported by Kroese et al in a recent work,^24^ survival outcomes are similar. Indeed, they report a median OS in patients local treatment alone of 24 months and in those receiving local plus systemic therapy of 35 months. On the contrary, PFS was significantly longer (16 and 28 months), representing an excellent results may be linked with a strict patients’ selection.

We must acknowledge the limitations of the present study. First of all, this is a retrospective analysis, so selection biases cannot be ruled out. It is likely that the patients represent a selected subpopulation of the whole Stage IV scenario; therefore the good survival rates could at least partially reflect this selection. However, we should highlight the good homogeneity of our series, since all patients were selected for histology, and also how our results are in line with available literature. Also number of patients in our study is quite low; however, this still represents one of the larger published experiences and the only one, at the best of our knowledge, focusing on adenocarcinoma. Lastly, heterogeneity of disease stage, treatments received and SRT dose and fractionations represents a further limitation particularly for the identification of prognostic factors.

## Conclusion

SRT is a valid, non-invasive and non-toxic treatment for OMD in esophagogastric adenocarcinoma. Survival outcomes were satisfactory, although further distant failure still represents a major challenge. Response of the irradiated lesion(s) correlated with better OS and PFS, highlighting the opportunity of an ablative local approach in these patients. Further research is necessary to better define the role of local approaches and to better integrate them with systemic therapies.
